# Spinosad 0.9% Lotion for Scabies on Skin of Color: An Interventional Study by Polarized and UV-Induced Fluorescence Dermoscopy

**DOI:** 10.7759/cureus.107968

**Published:** 2026-04-29

**Authors:** Balachandra S Ankad, Srishti R Patil, Deepanshu P Bansal

**Affiliations:** 1 Department of Dermatology, Shri Nijalingappa Medical College, Bagalkot, IND

**Keywords:** dermoscopy, greenish fluorescence, mite, scabies, spinosad, treatment, triangular delta sign

## Abstract

Introduction

Scabies, caused by *Sarcoptes scabiei var. hominis*, is a highly prevalent ectoparasitic infestation and a recognized neglected tropical disease. Increasing incidence, persistent post-treatment symptoms, and emerging resistance to conventional therapies such as permethrin necessitate effective alternative treatments. Spinosad 0.9% lotion, with known ovicidal and antiparasitic properties, has recently gained attention. This study evaluates its therapeutic efficacy using clinical and dermoscopic parameters, including polarized and ultraviolet-induced fluorescence (UVF) imaging.

Materials and methods

A hospital-based interventional case series was conducted at a tertiary care center from December 2025 to January 2026. Forty clinically and dermoscopically confirmed scabies patients were enrolled. Baseline demographic, clinical, and dermoscopic findings were recorded. Dermoscopy was performed using polarized and UVF modes to detect mites and burrows. Patients were treated with a single overnight application of spinosad 0.9% lotion. Follow-up assessment at three weeks included evaluation of clinical lesions (papules, nodules, pustules, excoriations) and dermoscopic signs (triangular jet sign, curvilinear tracks, and fluorescence). Statistical analysis was performed using SPSS, with p<0.05 considered significant.

Results

The study population had a mean age of 22±13.33 years, with a male predominance. Papules were the commonest lesions. Significant reduction was observed in papules (from 37, 92.5%, to five, 20%) with p<0.05; nodules (from 16, 40%, to one, 2.5%) with p<0.01; and pustules (from five, 12.5%, to none) with p<0.05 after treatment. Excoriations showed no significant change. Dermoscopic detection of mites decreased from 29 (72.5%) at baseline to nine (22.5%) at follow-up (p<0.05). Both polarized and UVF dermoscopy effectively demonstrated a reduction in mite-related features. No adverse effects were reported.

Discussion

Spinosad demonstrated significant clinical and dermoscopic efficacy, consistent with previous studies, and offers advantages such as single application and ovicidal activity. Persistent excoriations likely reflect ongoing post-scabetic pruritus rather than treatment failure. Dermoscopy proved to be a valuable objective tool for monitoring therapeutic response.

Conclusion

Spinosad 0.9% lotion is an effective, safe, and convenient alternative for scabies treatment, particularly in settings of resistance or poor compliance. Dermoscopic evaluation enhances diagnostic accuracy and treatment monitoring.

## Introduction

Scabies is a highly prevalent ectoparasitic infestation caused by the mite *Sarcoptes scabiei var. hominis*, and it continues to pose a significant public health concern, particularly in developing countries. The mite burrows into the epidermis, and after an incubation period of 2-3 weeks, induces intense pruritus, which is typically more severe during nighttime [1]. In 2017, the World Health Organization classified scabies as a neglected tropical disease due to its ease of transmission within communities, frequent outbreaks, long-term morbidity, and associated economic burden [2].

Dermoscopy has emerged as a reliable diagnostic tool in scabies, enabling visualization of the mite, its burrows, and related products such as eggs and fecal matter. Both polarized dermoscopy and ultraviolet-induced fluorescence (UVF) modes are useful for detecting the mite [3].

Currently, permethrin 5% cream or lotion and oral ivermectin are considered the treatments of choice for scabies [4]. However, dermatologists in India have been encountering a marked increase in scabies cases in recent years. Despite appropriate therapy, persistence of pruritus and skin lesions after treatment is common, often leading to diagnostic uncertainty, unnecessary retreatment, and prolonged patient distress. Therefore, there is a critical need for effective alternative therapies.

Spinosad, a neurotoxic insecticide derived from the fermentation of *Saccharopolyspora spinosa*, has recently gained attention as a topical antiparasitic agent due to its favorable safety profile and ovicidal activity [5]. In this context, the present study evaluates the efficacy of spinosad 0.9% lotion in scabies, with therapeutic assessment using both polarized and UVF dermoscopy.

## Materials and methods

It was a hospital-based interventional case series study conducted at a tertiary care hospital attached to Shri Nijalingappa Medical College, Bagalkot, from December 2025 to January 2026. Institutional ethical approval (SNMC/IECHSR/2026/A-3/1) was obtained, and informed consent was secured from all patients. Clinically diagnosed and dermoscopically confirmed cases of scabies were consecutively enrolled for evaluation. Inclusion criteria were treatment-naïve patients who had not received any prior therapy for scabies, and patients who had received treatment for scabies at least three months prior to participation in the study. Exclusion criteria were patients presenting with lesions complicated by secondary infection and patients who had received any treatment for scabies within three months prior to enrolment in the study. Demographic details, including age, sex, occupation, and duration of disease, were recorded. Appropriate clinical photographs were taken. Dermoscopic examination was performed using a DL5 dermoscope at 10× magnification in both polarized and UVF modes, and images were captured using a smartphone.

Patients were instructed to apply spinosad 0.9% lotion over the entire body, from the neck down to the toes, at night. They were advised not to wash or bathe for at least six hours after application and not to repeat the application. An antihistamine was prescribed to alleviate pruritus. Patients were reassessed after three weeks of therapy based on clinical criteria (pruritus, papules, nodules, and pustules) and dermoscopic criteria, including the presence or absence of mites (triangular delta sign), white curvilinear tracks, and greenish fluorescence.

The collected data were entered into Microsoft Excel (Microsoft Corp., Redmond, US) and analyzed using IBM SPSS Statistics version 25.0 (IBM Corp., Armonk, US). Qualitative variables were expressed as frequencies and percentages, while quantitative variables were summarized as mean±standard deviation or median with interquartile range, as appropriate. Differences in clinical lesions and dermoscopic findings before and after treatment were assessed using McNemar's chi-square test. A p-value <0.05 was considered statistically significant, and a p-value <0.01 was considered highly statistically significant.

## Results

The study included 40 patients (25 males and 15 females), with a mean age of 23.5±13.43 years. The majority of patients belonged to the 10-30-year age group. The median duration of symptoms was 20 days (interquartile range: 15-30 days). The most common site of involvement was the trunk, observed in 35 (87.5%) of patients (see Table 1).

**Table 1:** Epidemiological and clinical characteristics of patients diagnosed with scabies

Variables	Category	n	%
Age	1-10	7	17.5
	11-20	11	27.5
	21-30	11	27.5
	31-40	7	17.5
	41-50	2	5.0
	51-60	2	5.0
	Mean±SD	23.5±13.43	
Sex	Male	25	62.5
	Female	15	37.5
Duration	≤10 days	6	15
	10-20 days	18	45
	20-30 days	11	27.5
	>30 days	5	12.5
	Median (IQR)	20 (15, 30)	
Sites	Folds	20	50
	Web space	23	57.5
	Trunk	35	87.5
	Genitalia	26	65
	Limbs	31	77.5

**Figure 1:**
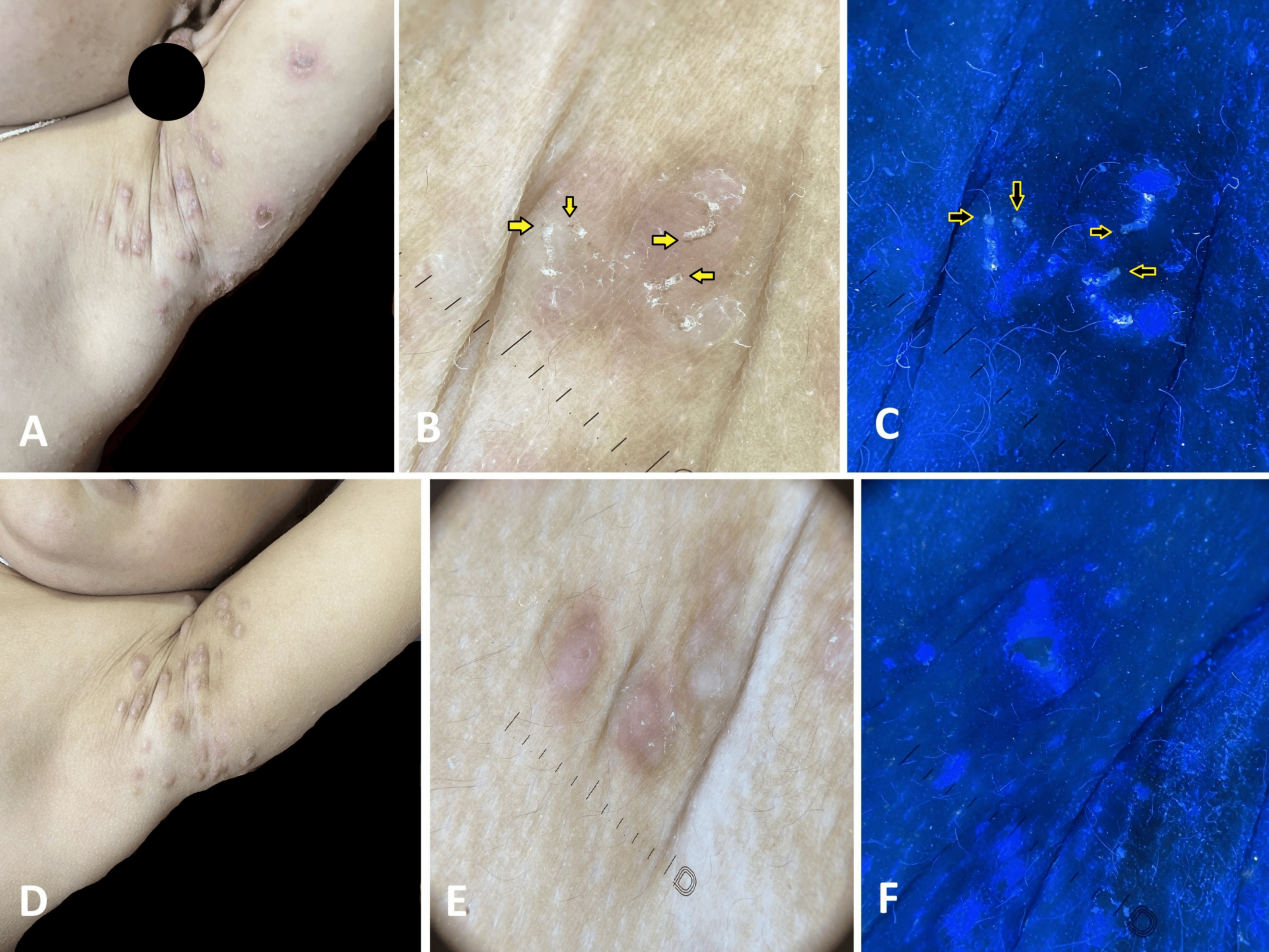
A two-year-old boy suffering from scabies A: Pre-treatment clinical image shows multiple nodules and excoriations in the axilla. B: Polarised dermoscopy shows triangular delta sign (mite; yellow arrows), curvilinear white tract (burrow), and pink areas. C: UVF mode shows greenish fluorescence (mite; black arrows). D: Post-treatment clinical image reveals absence of excoriations and reduction in the size of nodules. E: Polarised dermoscopy shows pinkish areas without signs of mite. F: UVF mode shows absence of greenish fluorescence. DermLite® DL5, 10xmagnification UVF - ultraviolet-induced fluorescence

**Figure 2:**
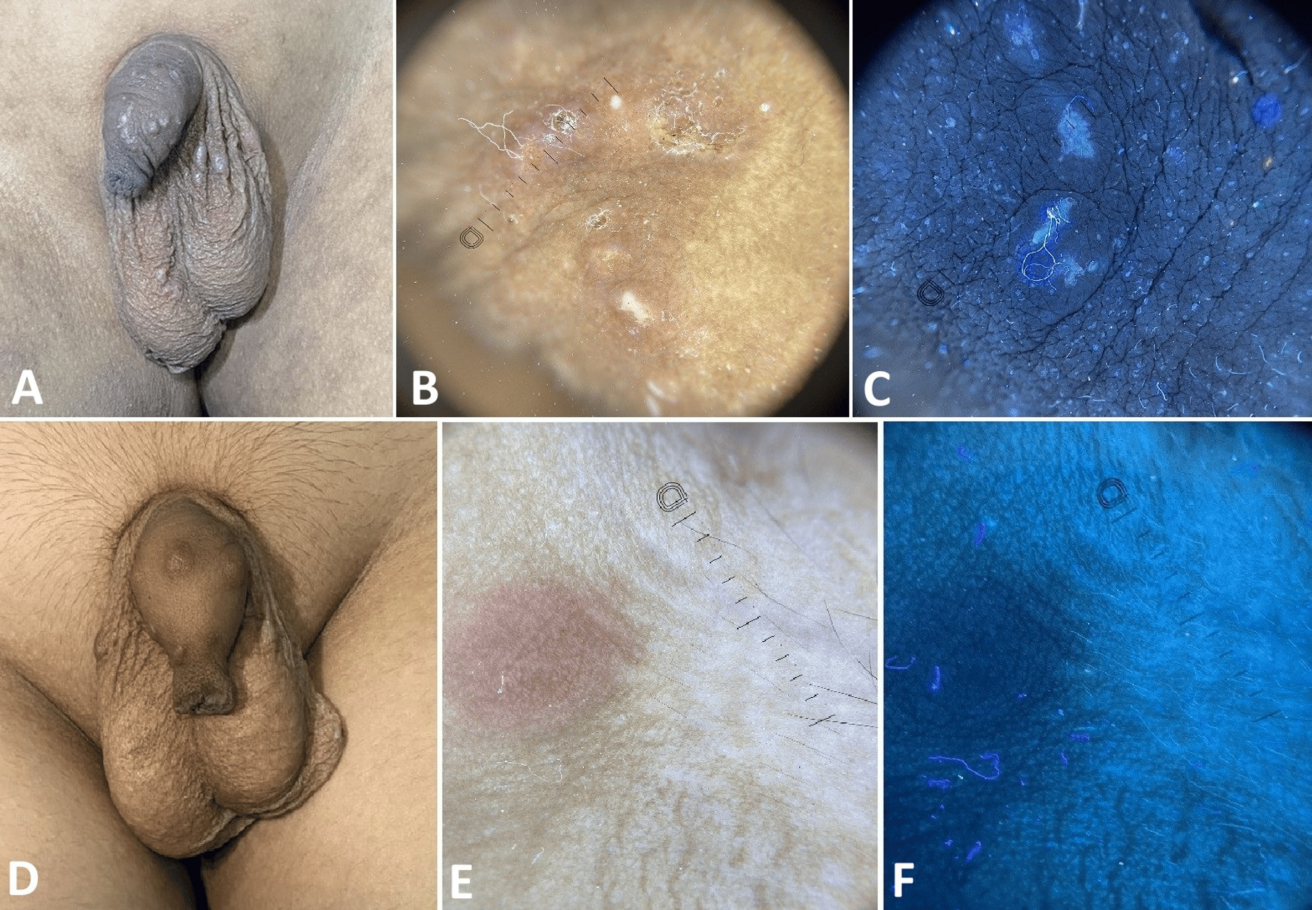
A ten-year-old patient suffering from scabies A: Pre-treatment clinical image shows multiple papules and pustules on the genitalia. B: Polarised dermoscopy shows yellowish-white areas (pustules) and pink areas. C: UVF mode shows bluish-white fluorescence. D: Post-treatment clinical image reveals few papules. E: Polarised dermoscopy shows a pinkish area. F: UVF mode reveals the black area. DermLite® DL5, 10xmagnification UVF - ultraviolet-induced fluorescence

**Figure 3:**
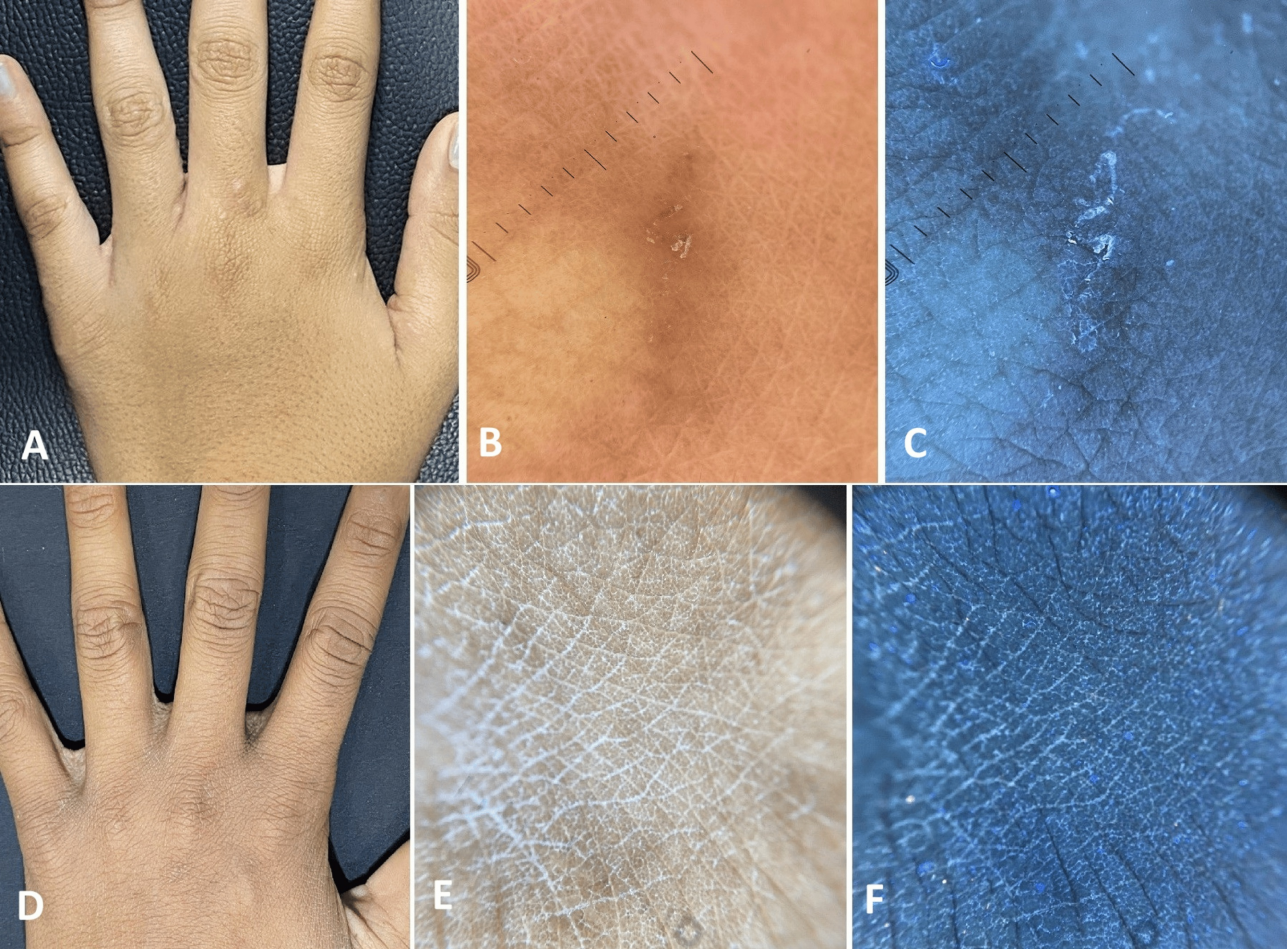
A twelve-year-old female patient affected by scabies A: Pre-treatment clinical image shows multiple papules in the finger web spaces. B: Polarised dermoscopy shows curvilinear white tracts and a reddish-brown area. C: UVF mode shows bluish-white fluorescence. D: Post-treatment clinical image reveals clearance of papules. E: Polarised dermoscopy shows the absence of white tracts. F: UVF mode reveals absence of fluorescence. DermLite® DL5, 10xmagnification UVF - ultraviolet-induced fluorescence

**Figure 4:**
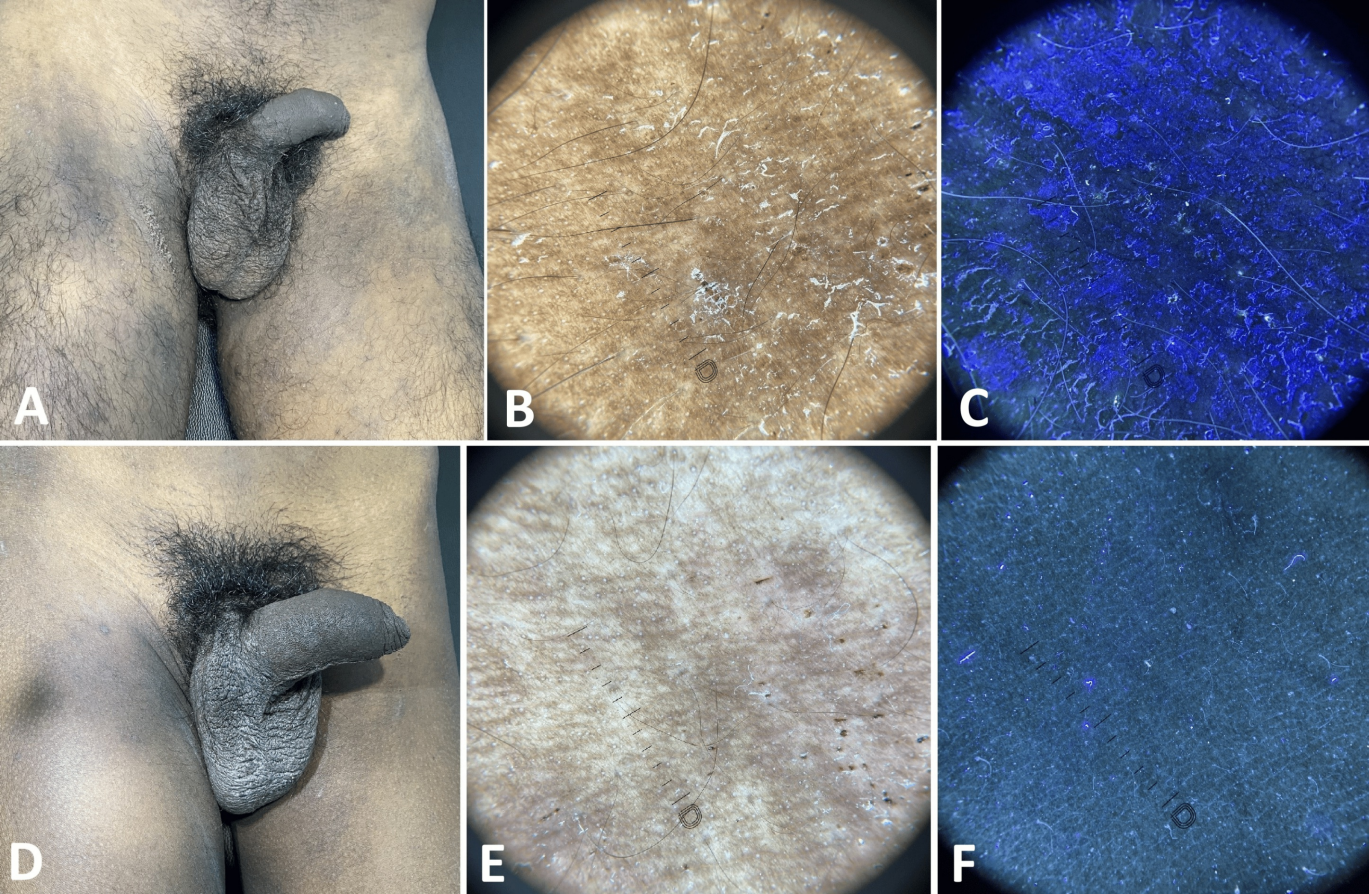
A twenty-year-old patient suffering from scabies affecting genitalia and thighs A: Pre-treatment clinical image shows multiple excoriation on the inner thigh scrotum. B: Polarised dermoscopy shows reddish-brown areas and white scales. C: UVF mode shows bluish-white fluorescence. D: Post-treatment clinical image reveals clearance of scales and a few excoriations. E: Polarised dermoscopy shows a few reddish-brown dots. F: UVF mode reveals the absence of fluorescence. DermLite® DL5, 10xmagnification UVF - ultraviolet-induced fluorescence

At baseline visit, clinically, nodules (Figure 1A), papules (Figures 2A, 3A), pustules (Figure 2A), and excoriations (Figure 4A) were observed with varying frequencies, with papules being the most common lesions in 37 (92.5%) patients. Mites were detected in 29 (72.5%) patients at baseline, which decreased to nine (22.5%) patients after treatment. The presence of mite and burrows was identified respectively as triangular delta sign (Figure 1B) and as curvilinear white tracts (Figures 1B, 3B) in polarised light, whereas in UVF mode they were found as greenish fluorescence (Figure 1C) and bluish-white fluorescence (Figures 1C, 3C). Nodules and papules were observed as pinkish areas (Figures 1B, 2B), and pustules were seen as yellowish-white areas (Figure 2B) in polarised light. Excoriations were represented by reddish-brown areas and white scales (Figure 4B) in polarised mode and bluish-white fluorescence in UVF mode (Figure 4C).

Dermoscopy demonstrated decreased frequency of mite after treatment. Absence of triangular delta sign (Figure 1E), curvilinear white line (Figures 1E, 3E), reduction in the pink areas (Figures 1E, 2E), reddish-brown areas (Figures 3E, 4E), and white scales (Figure 4E) were noted in polarised mode. UVF mode revealed the absence of bluish-white fluorescence due to burrow and scale (Figures 1F, 2F, 3F, 4F). 

As shown in Table 2, following treatment, there was a statistically significant reduction in the size of the nodules (p<0.01; Figure 1D), papules (p<0.05; Figures 2D, 3D), and pustules (p<0.05; Figure 2D). However, no significant change was noted in the frequency of excoriations (p=0.2278 Figure 4D).

**Table 2:** Changes in clinical features following treatment with spinosad 0.9% lotion

Clinical lesions	Before treatment n (%)	After treatment n (%)	Chi-square value	p-value
Papules	37 (92.5)	8 (20)	43.6	<0.05
Excoriation	30 (75)	25 (62.5)	1.29	0.2278
Nodule	16 (40)	1 (2.5)	17.3	<0.01
Pustule	5 (12.5)	0 (0)	5.13	<0.05

The presence or absence of mites was assessed using dermoscopy. The comparison between pre- and post-treatment findings demonstrated a statistically significant difference (two-tailed p=0.0072). According to conventional criteria, this result is considered highly significant. The p-value was calculated using McNemar's chi-square test with continuity correction (χ²=7.225, df=1; see Table 3).

**Table 3:** Dermoscopic evaluation of treatment response in scabies

Dermoscopy	Before treatment n (%)	After treatment n (%)	McNemar's chi-square test value	p-value
Mite present	29 (72.5)	9 (22.5)	7.225	<0.0072
Mite absent	11 (27.5)	31 (77.5)		

## Discussion

Scabies affects more than 200 million people annually and is characterized by intense nocturnal itching that disrupts sleep and daily activities. This can significantly impact quality of life, particularly in school-going children, leading to reduced concentration and impaired academic performance [6,7].

The current drug of choice is permethrin 5% cream, which is applied to the entire body below the neck and repeated after one week. Other topical agents include 0.5% malathion in an aqueous base and 10-25% benzyl benzoate. However, resistance to permethrin has been increasingly reported. This resistance is attributed to mutations in voltage-gated sodium channels (VGSCs), which reduce permethrin binding, and increased activity of glutathione S-transferase (GST), which enhances drug detoxification. Additionally, treatment failure may occur due to improper application or failure to repeat treatment after 7-14 days; this phenomenon is referred to as pseudo-resistance [8]. Given these challenges, there is a need for effective alternative therapies, particularly in countries like India, where overcrowding, low socioeconomic conditions, and poor treatment compliance are prevalent.

Spinosad 0.9% lotion has been evaluated as a potential alternative in a study by Seiler et al. [5]. In their study, the authors compared the efficacy of spinosad lotion with its vehicle and observed a complete cure in the spinosad group. Clinical, dermoscopic, or microscopic assessments were used for evaluation. The treatment demonstrated a favorable safety profile, with only a few subjects reporting transient burning sensations. Similarly, in the present study, patients showed statistically significant improvement in nodules (p<0.01), pustules (p<0.05), and papules (p<0.05). Dermoscopic evaluation revealed a significant reduction in signs of mite, such as the triangular delta sign under polarized mode and greenish fluorescence under UVF mode (p<0.05). No adverse effects were reported.

Spinosad is derived from the soil actinobacterium* Saccharopolyspora spinosa* and functions as an insecticide. Preclinical studies have demonstrated that it is poorly absorbed through the skin, non-allergenic, and non-genotoxic. It was approved by the United States Food and Drug Administration in January 2011 for the treatment of head lice infestation as a 0.9% suspension [9,10].

In the present study, spinosad was used in lotion form (Nuper Therapeutics, India). The study population included patients with skin of color, and treatment response was objectively assessed using dermoscopy, a well-established diagnostic tool for scabies [11,12]. Following treatment, nodules, papules, and pustules showed marked improvement in nearly all cases. However, excoriations and nodular lesions persisted in some patients, indicating that post-treatment pruritus may continue for 4-6 weeks and granulomatous infiltrate in nodular lesions. Dermoscopic signs of mite and burrow were significantly reduced.

Given the increasing incidence of scabies globally and in India, spinosad represents a promising therapeutic option [6,13]. Overall, findings from both previous studies and the present study suggest that spinosad is an effective alternative in cases of permethrin resistance. Hence, it is recommended to use spinosad for scabies.

This study has limitations; the sample size was small, and no comparison was made between spinosad and standard treatments for scabies. Objective scoring systems to assess the efficacy of treatment on clinical lesions and pruritus were not utilized. As the study was conducted in a tertiary referral care center, the findings may not be generalizable to the broader community. Therefore, further studies with larger sample sizes are recommended to validate these results.

## Conclusions

Spinosad 0.9% lotion, now available in India, is an effective alternative for the treatment of scabies. It does not penetrate the deeper layers of the skin and is non-genotoxic, contributing to its favorable safety profile. The convenience of a single application, along with the minimal incidence of adverse effects, makes it an excellent therapeutic option. Additionally, dermoscopy serves as a useful and objective tool for assessing treatment response.
